# Epidemiology of *Giardia duodenalis* infection in ruminant livestock and children in the Ismailia province of Egypt: insights by genetic characterization

**DOI:** 10.1186/1756-3305-7-321

**Published:** 2014-07-11

**Authors:** Yosra A Helmy, Christian Klotz, Hendrik Wilking, Jürgen Krücken, Karsten Nöckler, Georg Von Samson-Himmelstjerna, Karl-H Zessin, Toni Aebischer

**Affiliations:** 1 Department of Animal Hygiene, Zoonoses and Animal Ethology, Faculty of Veterinary Medicine, Suez Canal University, 41511 Ismailia, Egypt; 2 Faculty Panel Veterinary Public Health, Freie Universität Berlin, 14163 Berlin, Germany; 3 Mycotic and parasitic agents and mycobacteria, Department of Infectious Diseases, Robert Koch-Institute, 13353 Berlin, Germany; 4 Gastrointestinal Infections, Zoonoses and Tropical Infections, Department of Infectious Disease Epidemiology, Robert Koch-Institute, 13353 Berlin, Germany; 5 Institute for Parasitology and Tropical Veterinary Medicine, Freie Universität Berlin, 14163 Berlin, Germany; 6 Federal Institute for Risk Assessment (BfR), 12277 Berlin, Germany

**Keywords:** *Giardia*, Genetic characterization, Ruminants, Diarrheal children, Real time PCR, *RIDA^®^QUICK* test, Triose phosphate isomerase (*tpi*), Glutamate dehydrogenase (*gdh*), β-giardin (*bg*), Spatial distribution, Egypt

## Abstract

**Background:**

*Giardia duodenalis* is a common flagellated protozoan parasite that infects the small intestine of a wide range of vertebrate hosts. This study aimed to determine whether tracing of *G. duodenalis* isolates by current genetic typing tools is possible using an exemplary set of samples from infected cattle, buffalo and children from the Ismailia province, Egypt.

**Method:**

A total of 804 fecal samples from ruminant animals was collected from 191 herds and 165 samples from diarrheal children below the age of 10 years. Parasites were detected in these samples using the copro-antigen *RIDA^®^QUICK* test and by real-time PCR. Samples were then genetically characterized based on the triosephosphate isomerase, glutamate dehydrogenase and β-giardin genes.

**Results:**

The prevalence of *G. duodenalis* was 53% in ruminants and 21% in symptomatic children and infection was not positively correlated with diarrheal symptoms. Sequence typing analysis confirmed predominance of B-type sequences (>67%) in humans and E-type sequences (>81%) in ruminants over A-type sequences. For 39 samples the complete sequence information of the three marker gene fragments could be derived. Integration of the concatenated sequence information of the three marker gene fragments with the spatial data of the respective sample revealed that identical or near identical (only up to 1 out of 1358 bp different) concatenated sequencing types were spatially related in 4 out of 5 cases.

**Conclusion:**

The risk of zoonotic infection emanating from ruminants even in high prevalence areas is negligible. Genetic characterization indicated a predominant anthropogenic cycle of infection within the pediatric population studied. Integration of sequence typing data with information on geographic origins of samples allows parasite sub-population tracing using current typing tools.

## Background

*Giardia duodenalis* (syn. *G. lamblia* and *G. intestinalis*) is one of the most frequent enteroparasites worldwide and has been included in the WHO ‘neglected disease initiative’ [1]. In particular, children in resource-poor countries can be severely affected by *G. duodenalis* infections, which may lead to significant malabsorption, weight loss and growth retardation [1,2]. The gastrointestinal manifestations include diarrhea, abdominal cramps, greasy stools, flatulence, epigastric tenderness, and steatorrhea accompanied by full-blown malabsorption syndrome [3]. However, infection that is not associated with such overt symptoms occurs frequently, especially in resource-poor countries [4]. These infections may often go unnoticed but epidemiological observations suggest that they are still associated with a malabsorption phenotype [5].

Transmission of *Giardia* cysts to humans occurs mainly via ingestion of contaminated water or food but parasites can also be directly contracted from infected individuals [6]. In resource-poor countries *Giardia* is still mainly diagnosed by light microscopy and detection of either trophozoites or cysts in fecal samples. This technique suffers from low sensitivity in comparison to improved methods based on immunodiagnostic fecal antigen detection or polymerase chain reaction (PCR) [7-9]. The latter assays are thus more useful for epidemiological studies [5,9,10], in particular in combination with genetic characterization of the parasites [6]. It is well established that *G. duodenalis* represents a species complex composed of at least eight genetic groups (assemblages A to H) that are distinguishable based on genetic polymorphisms in, e.g., the triosephosphate isomerase (*tpi*), glutamate dehydrogenase (*gdh*), beta-giardin (*bg*) and small-subunit rDNA (ssu-rDNA) genes [6,11-15]. Typing according to these markers is relatively robust and to date has revealed limited evidence for inter-assemblage recombination [16]. If it did occur, such recombination would likely have a tremendous effect on parasite host ranges since assemblages differ in their host specificity, with assemblages A and B showing the broadest host range infecting humans and numerous other mammals including companion animals and livestock [15,17-19]. *G. duodenalis* is considered potentially zoonotic, largely due to the host distribution of A- and B-type parasites [15,18,19]. However, the impact, if any, of zoonotic transmission in countries with low or high prevalence remains unclear [15].

The scope of the present study was to improve the understanding of the epidemiology of *G. duodenalis* infections in the Ismailia province in Egypt. Therefore, the prevalence of *Giardia* infections in children with diarrheal symptoms and in ruminants (cattle and buffalo) was determined. Infection was assessed using an immunodiagnostic, copro-antigen detection test (*RIDA^®^QUICK)* and a real time PCR- assay for detection of *Giardia* ssu-rDNA. In addition, parasite DNA was genetically characterized based on *tpi*, *gdh* and *bg* genes to elucidate distribution of *G. duodenalis* types, and to gather evidence for transmission of *Giardia* from ruminants to humans in this area.

## Methods

### Ethics statement

The study was reviewed and approved by the University Board, Suez Canal University. Approval included the use of oral consent (human and animal samples) due to low literacy rates. Oral consent of the parents or legal guardians of the children was obtained in each case and was witnessed by a hospital physician or chief nurse. Privacy and confidentiality were assured. The oral consent was documented in the patient’s medical record in the hospital. All samples were non-invasive (only fecal samples) and were analyzed anonymously. Fecal samples from animals were collected rectally after approval from their owners and assurance of anonymity, witnessed by a veterinarian from the Egyptian Veterinary Medicine Authority.

### Study area and sample collection

The study area was the Ismailia province of Egypt from April to June 2011. The Ismailia arable belt runs along the Suez Canal and is one of the most densely populated provinces in Egypt with regard to livestock and people [20]. For 98% of the farms, farmers either do not own agricultural land or holdings are very small (<0.4 ha; herd sizes <10; low producing native or crossbred animals) with mixed herds of cattle and buffalo. Animals in the smallholder system are kept in the home compounds and are fed with crop by-products. They have access to surface water or water from shallow wells [20].

The survey considered the distribution of animal holdings over the districts of the province, and aimed to collect fecal samples from animals of a proportional number of the predominantly smallholder farms per district. Samples were mixed with an equal amount of 5% potassium dichromate and kept at 4°C until examination.

A total of 804 animal fecal samples were collected from 191 herds, 593 samples from cattle (246 males and 347 females) and 211 samples from buffalo (83 males and 128 females). Sampling included diarrheal and non-diarrheal animals, with an emphasis on calves. Each animal was identified using a specific system: nomenclature consists of the herd followed by the animal number. For example, 31b/C7 represents cattle 7 (C for cattle, B for buffalo) of the herd b at location 31.

Concurrently, stool samples from 165 children up to 10 years of age were collected [20]. Of these, 136 were from diarrheal children hospitalized in one of the province’s 9 district hospitals, with 10 to 23 stool samples per hospital. 29 were from diarrheal children living on farms where animal samples were also collected.

Attributes of animals (type of drinking water and type of soil available) and children (type of drinking water, place of residence and animal contact) were recorded. The prevalence of *Cryptosporidium* spp. in this sample population was reported in an earlier study [20].

### Giardia diagnostics

All samples were examined using the copro-antigen *RIDA^®^QUICK Cryptosporidium/Giardia* Combi (N1122) kit (R-BIOPHARM AG, Darmstadt, Germany) as a screening test, following the manufacturer’s instructions with 100 μl of the liquid fecal sample. According to the information provided by the supplier, the screening test (for fresh human samples) has 100% sensitivity, 95.2% specificity, 88.2% positive predictive value, and 100% negative predictive value compared to conventional microscopy that was used as gold standard.

Animal samples that were positive in the *RIDA^®^QUICK* test and 10% of randomly selected negative samples [21] were tested further using *Giardia*-specific real-time PCR [22]. All human samples were subjected to *RIDA^®^QUICK* test and *Giardia*-specific real-time PCR. For the *Giardia*-specific real-time PCR a previously established protocol was applied [9,22] using the Maxima Probe qPCR Master Mix (Thermo Scientific) with 1 μl of the target DNA in a total volume of 25 μl. Amplification (40 PCR cycles), detection, and data analysis were performed using an Mx3000P cycler and MxPro software (Stratagene). Analytical specificity was 94.1% (1 of 17 non-template-controls was weakly positive, ct = 38.9). Analytical sensitivity was determined using a calibrated parasite DNA solution and was equivalent to the detection of 1 parasite (1 trophozoite = 4n) giving a ct = 39.8.

### DNA extraction

Genomic DNA was extracted from 1 ml of sample using the NucleoSpin^®^ Soil kit (Macherey-Nagel, Germany) and using an elution volume of 60 μl. Extracted DNA was analyzed by agarose gel electrophoresis and stored at -20°C until further examinations. DNA concentration and purity were assessed spectrophotometrically.

### Genetic characterization of Giardia isolates

Genetic characterization of *Giardia* isolates was performed using previously described nested PCR protocols to amplify fragments of the *tpi-*[13], *gdh*[12]- and *bg*[14,23]-gene loci. Some of the samples were also analyzed for assemblage A- and E-specific *tpi*-gene fragments as previously published [24]. As a standard procedure, 1–3 μl of sample DNA were used for the primary PCR and 1–2 μl of the primary PCR reaction were applied to the nested PCR. All PCR reactions were performed using 2.5 U of the MangoTaq™ DNA Polymerase and the according buffer system (Bioline).

PCR reactions were analyzed by agarose gel electrophoresis and PCR products were purified using the DNA Clean&Concentrator™ kit (ZYMO RESEARCH). Sequencing reactions were performed using Big dye 3.1 sequencing reagents (Applied biosystems) and applied to the RKI in-house sequencing facility. The obtained sequences were analyzed to identify the most similar sequence deposited in public databases by applying the Basic Local Alignment Search Tool (BLAST) (http://blast.ncbi.nlm.nih.gov/Blast.cgi) using the nucleotide collection (nr/nt) database in April 2013. The sequences were also compared to dedicated reference sequences [25] in order to identify the assemblage and sub-assemblage level. Therefore, alignments of single and in silico concatenated genes were produced using MUSCLE [26] or ClustalW integrated subroutines of Geneious version 6.1.6 (Biomatters). Sequence relatedness was analyzed by distance comparison between sequences based on the number of identical nucleotide residues. See Additional file 1: Table S1 for the accession numbers of the reference sequences used for the analysis. For spatial analysis sequences that were identical or that differed only in one nucleotide were combined in ‘clusters’ and sample distribution was visualized onto the map of the Ismailia province using the program ArcGIS 9.5 (ESRI).

### Nucleotide sequence accession numbers

Nucleotide sequences generated in this study have been deposited into the GenBank database under accession numbers [GenBank: KF957627-KF957634, KJ124862-KJ124918, KJ124983-KJ125044, KJ124919-KJ124982].

### Statistical analysis

Statistical analysis was performed using mid-P exact probability tests and differences were considered significant when *p*-values ≤ 0.05 were obtained (http://www.openepi.com/v37/Menu/OE_Menu.htm). The real-time PCR-based *Giardia* prevalence in animal samples was estimated by re-testing a random selection of 10% of samples negative in the rapid screening test and extrapolating the data to the whole animal sample population [21].

## Results

### Estimation of the prevalence of Giardia infections in humans and accompanying livestock

Copro-antigen tests are attractive for epidemiological surveys of the burden of gastrointestinal infections since they are highly processive. Here the *RIDA^®^QUICK* test was used to quickly evaluate *Giardia* prevalence in all samples collected for this survey. In total, 13 out of 165 (8%) samples from diarrheal children and 50 out of 804 (6%) animal samples tested positive. However, based solely on antigen-detection tests, prevalence can be significantly underestimated (although less than when based on classical microscopic tests; [5,7,27]). Therefore, a more sensitive strategy based on a real-time PCR assay [9,22] directed to the multi-copy parasite ssu-rDNA in fecal samples was designed to obtain a more accurate estimate of the prevalence. To this end, all human samples were re-analyzed and, to estimate prevalence in animals, samples of all copro-antigen test positive ruminants were re-analyzed as well as a randomly chosen 10% of the copro-antigen test negative animal samples.

In children with diarrhea, results from real-time PCR revealed an overall *Giardia* prevalence of 21% (Table 1). The age dependent prevalence varied but the highest value (29%) was observed in children up to 1 year old (not significant). Interestingly, the real-time PCR assays on samples from these infants yielded comparatively high ct values indicating lower parasite DNA quantities relative to samples from older children (Table 1). All samples in this infant age group that were tested positive by real-time PCR were negative in the copro-antigen test. A comparison of median real-time PCR ct values from samples positive in both the copro-antigen test and by real-time PCR, versus samples only positive by real time PCR suggested a significantly higher abundance (~120 fold) of *Giardia* DNA in copro-antigen test-positive samples (median ct = 28.1 vs. = 35.1, p <0.05, Mann Whitney test).

**Table 1:** **Estimation of *Giardia duodenalis *** **prevalence in diarrheal children**

	***Giardia *** **prevalence in percent**	
	**(Number of samples: positive/total)**	
	**(95% confidence interval)**	
**Age group**	**Copro-antigen test (*RIDA^®^QUICK)***	**Real time PCR assay**
up to 1y	0	29
	(0/24)	(7/24)
	(0–11.7)	(13.7–49.4)
		Ct value: 38.4 (33.2 – 39.0)
>1y-2y	9	26
	(3/35)	(9/35)
	(2.2–21.6)	(13.3–41.9)
		Ct value: 34.5 (26.1 – 38.0)
>2y-3y	6	16
	(2/32)	(5/32)
	(1.1–19.2)	(5.9–31.3)
		Ct value: 30.7 (18.7 – 37.4)
>3y-4y	10	24
	(2/21)	(5/21)
	(1.6–28.1)	(9.3–45.2)
		Ct value: 33.8 (29.3 – 39.3)
>4y-10y	11	17
	(6/53)	(9/53)
	(4.7–22.1)	(8.6–28.9)
		Ct value: 29.0 (22.7 – 39.6)
Total	8^a^	21^b^
	(13/165)	(35/165)
	(4.5–12.8)	(15.5–28)
		Ct value: 33.2 (18.7 – 39.6)

Different superscripts (a, b): significant difference at p < 0.05.

Median ct values (range) of real time PCR assay for assigned age groups are shown.

In animals, all positive copro-antigen tests were confirmed by real-time PCR. In addition, 50% of the subset of copro-antigen negative samples were also positive. Thus, a value for total prevalence of 53% in the sampled livestock was extrapolated (Table 2). The age-specific prevalence estimate ranged between 50-57%, with a trend for higher values in young calves (1 day-3 months, not significant) (Table 2). Moreover, testing positive for *Giardia* in either the copro-antigen or the real-time PCR based test was not correlated with diarrhea in these animals (data not shown and Table 3).

**Table 2:** **Estimation of *Giardia duodenalis *** **prevalence in cattle and buffalo**

	***Giardia *** **prevalence in percent**				
	**(Number of samples: positive/total)**				
	**(95% confidence interval)**				
	**Copro-antigen test (*RIDA^®^QUICK)***			**Real time PCR assay**^**b**^	
**Age group**	**All**	**Cattle**	**Buffalo**	**10% of quick test negative samples**	**Total estimated prevalence**
1d-1 m	9	9	8	52	57
	(22/244)	(17/183)	(5/61)	(12/23)	(138/244)
	(5.9–13.1)	(5.7–14.2)	(3.1–17.2)	(32.1–71.7)	(50.3–62.7)
>1-3 m	5	5	4	53	55
	(5/103)	(4/79)	(1/24)	(8/15)	(57/103)
	(1.8–10.4)	(1.6–11.8)	(0.2–18.9)	(28.7–76.8)	(45.7–64.7)
>4-6 m	6	7	2	46	50
	(9/149)	(8/107)	(1/42)	(6/13)	(74/149)
	(2.9–10.8)	(3.5–13.7)	(0.11–11.2)	(21.3–72.6)	(41.7–57.7)
>6 m	4	5	4	48	50
	(14/308)	(11/224)	(3/84)	(12/25)	(155/308)
	(2.6–7.3)	(2.6–8.4)	(0.9–9.4)	(29.2–67.3)	(44.8–55.9)
Total	6	7	5	50	53
	(50/804)	(40/593)	(10/211)	(38/76)	(424/804)
	(4.7–8.1)	(4.9–8.9)	(2.4–8.3)	(38.9–61.1)	(49.3–56.2)

^b^All copro-antigen test positives and 10% of the copro-antigen negative samples were analyzed by real time PCR. Prevalence was calculated by extrapolation assuming that the analyzed negative samples are representative for all negative samples. Note that all positive copro-antigen tests were confirmed by real time PCR assay.

Note: No significant differences were detected between the age groups.

**Table 3:** **Prevalence of *Giardia duodenalis *** **in animal samples in relation to fecal consistency**

	**Number of *Giardia *** **cases**							
	**(percent)**							
	**(95% confidence interval)**							
	***Copro-antigen (RIDA^®^QUICK) *** **test**				**Real time PCR assay**^**+**^			
	**Total**	**Watery**	**Pasty**	**Normal**	**Total**	**Watery**	**Pasty**	**Normal**
Positive	50	20	21	9	88	37	36	15
	(6)	(40*)	(42*)	(18*)	(70)	(42*)	(41*)	(17*)
	(5–8)	(27–54)	(29–56)	(9–30)	(61–77)	(32–53)	(31–51)	(10–26)
Negative	754	254	284	216	38	12	21	5
	(94)	(34*)	(38*)	(29*)	(30)	(32*)	(55*)	(13*)
	(92–95)	(30–37)	(34–41)	(26–32)	(22–38)	(18–48)	(39–70)	(5–27)
Total	804	274	305	225	126	49	57	20
		(34)	(38)	(28)		(39)	(45)	(16)
		(31–37)	(35–41)	(25–31)		(31–48)	(37–54)	(10–23)

^+^Only experimental results are presented.

*Percentage calculated as proportion of *Giardia* positive and negative samples, respectively.

Note: No significant differences of fecal consistency were detected of Giardia positive and Giardia negative animals.

### Risk factors for contracting Giardia infection

Occurrence of *Giardia* was analyzed for associations with potential risk factors determined for the two sampled populations. For humans, gender, residency status, farm animal contact, source of drinking water and co-infection with *Cryptosporidium* spp. (a second locally highly prevalent zoonotic protozoan that causes gastrointestinal disease) were investigated.

The prevalence of *Cryptosporidium* spp. infections amongst the studied cattle, buffalo and pediatric populations has been recently reported by our team [20]. Here, *Giardia-Cryptosporidium* spp. co-infections detected by real-time PCR were observed in 36% of animals and 11% of children. Assuming that co-infection will happen by chance, its prevalence can be estimated based on the product of the prevalence of individual infections. Accordingly, the expected prevalence of co-infection was calculated to be 17% in animals and 10% in children. Thus, the value in humans corresponded well with the observed numbers, suggesting neither positive nor negative effects of one infection on the risk of contracting the other pathogen. In animals, however, the calculated value was less than half of the observed prevalence (p <0.05, Fisher’s exact test) of co-infection implying a factor that promotes *Cryptosporidium* and *Giardia* co-infections.

For the pediatric population under study, gender, residency status and contact with livestock did not correlate with *Giardia* infection. However, a nearly 3-fold higher probability of *Giardia* infection was associated with drinking tap water (Table 4).

**Table 4:** **Analysis of potential risk factors for *Giardia *** **infections in diarrheal children**

	**Risk factor dependent *Giardia *** **infection in percent**							
	**(Number of *Giardia *** **cases: positive/total)**							
	**(95% confidence interval)**							
	**Gender**		**Residency**		**Contact with animals**		**Source of water**	
	**Male**	**Female**	**Village**	**City**	**Contact**	**No contact**	**Underground**	**Tap**
Copro-antigen test	9.5	6.2	9.0	7.0	9.0	5.0	5.0*	9.0*
	(8/84)	(5/81)	(7/82)	(6/83)	(10/108)	(3/57)	(3/57)	(10/108)
	(4.5–17.3)	(2.3–13.2)	(3.8–16.2)	(2.9-14.4)	(4.8–15.9)	(1.4–13.7)	(1.4–13.7)	(4.8–15.9)
Real time PCR assay	22.6	19.8	23.0	19.0	21.0	21.0	11.0^+^	27.0^+^
	(19/84)	(16/81)	(19/82)	(16/83)	(23/108)	(12/57)	(6/57)	(29/108)
	(14.6–32.5)	(12.2–29.5)	(15–33.2)	(11.9–28.8)	(14.4–29.8)	(11.9–33)	(4.4–20.6)	(19–35.8)

*p = 0.390; ^+^p = 0.013.

### Genetic characterization of Giardia suggests rare zoonotic transmission from livestock to humans

Copro-antigen detection and the chosen ssu-rDNA real-time PCR assay do not provide information about the sub-species or assemblage of *Giardia* present in a sample. The latter information, however, is necessary to assess likely transmission cycles. Thus*,* all samples that were applied to the real-time PCR assay were subjected to genetic characterization at the *tpi-, gdh-* and *bg*-gene loci [12-14]. Overall, parasite DNA from 90 samples (22 human and 78 animal samples) could be characterized at least at one locus (Additional file 2: Table S2). Of these, 52 samples could be typed at all three loci and 14 samples at two loci. Repeating the typing assays of the negative samples did not improve this yield (each locus-specific PCR was repeated at least once if a sample was negative in the first attempt). Genetic characterization yielded the following qualitative and quantitative results:

Firstly, typing produced previously observed ambiguities [6]. Typing of eight samples was ambiguous in the sense that sequences obtained for a particular locus or for different loci had to be assigned to different assemblages (Table 5). In another group of samples a different kind of expected ambiguity was observed, namely, double peaks at specific sites in the electropherograms of the sequencing reactions. Such peak patterns were observed in three assemblage A type, 15 B type and 39 E type sequences. Several of these patterns were repeatedly observed in different samples (Additional file 1: Table S1).

**Table 5:** **Samples assigning for different *G. duodenalis *** **assemblages at different gene loci**

**Sample ID**	**Host**	***tpi***	**Ass. A specific *tpi***	**Ass. E specific *tpi***	***bg***	***gdh***
6/C3	Cattle	A	A	-	E	E
42a/C3	Cattle	A	A	E	E	E
21b/C19	Cattle	E	-	-	E	A
19a/C3	Cattle	E	-	-	A	-
H135	Human	-	-	-	A/E	E
31a/C4	Cattle	A/E	-	E	E	E
H86	Human	B	A	-	B	A/B
15d/C10	Cattle	-	-	E	-	A/E

(-) = negative result.

Note: All PCRs and sequence analysis were performed at least twice with similar results.

Secondly, typing of *Giardia* DNA present in human samples confirmed that assemblage B is more frequently found than assemblage A (Table 6). Assemblage B was assigned based on *tpi* to 69%, on *gdh* to 67%, and on *bg* to 69% of the samples. Noteworthy, DNA from two human samples (samples H31 and H135) produced assemblage E type sequences at the *gdh* locus implying infection of children with a livestock assemblage (Table 6, Additional file 2: Table S2). For sample H135 results at the *gd* locus identified assemblage A in the sample most likely indicative of a mixed infection (Table 5). For H31 no other locus could be typed.

**Table 6:** **Type and number of *G. duodenalis *** **assemblages detected in animal and human samples by multi locus sequence typing**

**Assemblage**		**Animal**									**Human**		
		***Tpi***			***gdh***			***bg***			***tpi***	***gdh***	***bg***
		**Total**	**Cattle**	**Buffalo**	**Total**	**Cattle**	**Buffalo**	**Total**	**Cattle**	**Buffalo**			
*A*	A	8	6	2	1	1	-	1	1	-	-	1	2
	*AI*	1	1	-	-	-	-	-	-	-	-	-	-
	*AII*	1	1	-	-	-	-	-	-	-	5	2	2
*B*		-	-	-	-	-	-	-	-	-	11	12	11
*E*		46	40	6	51	43	8	46	39	7	-	2	-
*E/A*		1	1	-	1	1	-	-	-	-	-	-	1
*A/B*		-	-	-	-	-	-	-	-	-	-	1	-
Total		57	49	8	53	45	8	47	4	7	16	18	16

Thirdly, the dominance of assemblage E type sequences in ruminants was also confirmed [4,28] (Table 6). However, proportions assigned to assemblage E were variable depending on the locus analyzed (81% on the *tpi* locus, 94% on *gdh* and 98% on *bg* loci) (Table 6). Of note, typing efficiency of PCR at the different loci was 60% (*tpi*), 57% (*gdh*) and 51% (*bg*), respectively. Fourteen of 15 non-E type sequences deduced from animal samples belonged to assemblage A with 11 of these 14 detected by the *tpi* typing assay. Two of the A type *tpi* sequences found in animals (samples 10/C11 and 54b/C7, Additional file 2: Table S2) were identical to sub-assemblage AI and AII reference sequences, respectively. Interestingly, these were originally described for parasites isolated from human cases [17]. Unfortunately, complementing the typing of these samples at the other loci failed (Additional file 2: Table S2).

In summary, assemblage distribution within the host species investigated was consistent with expectations, as were assemblage- and locus-specific sequence polymorphisms [29].

### Relatedness and spatial distribution of sequencing types

Multi locus sequence typing data together with information on the geographic origin of isolates is increasingly used to understand the distribution and dynamics of pathogen populations or individual subgroups in space and time (c.f. http://www.spatialepidemiology.net/; [30]). Here, this approach was adopted to test if analysis of the genetic structure of the parasite population derived from the three loci typing scheme can yield epidemiologically relevant information. To this end, the potential relatedness of parasite DNA types obtained from human or animal samples was analyzed and then checked for its correspondence to the spatial distribution of the respective samples.

Full sequencing information with complete sequence of both strands of PCR fragments from all three loci was obtained for 43 samples. Four of these samples were excluded from the analysis because they contained single gene typing fragments assigned to different assemblages (see above) and were most likely reflecting mixed infections. Thus, for 39 samples sequences of the individual typing fragments were first trimmed to a common length and then concatenated in the gene order ^5’^-*tpi-bg-gdh*^3’^ to yield a total sequence length of 1358 bp. These concatenated sequences and reference concatenated sequences representing assemblage AI, AII, BIII, BIV, and E types (see Additional file 1: Table S1 for accession numbers of the respective type sequences) were subsequently aligned. Comparisons were then performed to derive the number of common or different base pairs for each concatenated sequence pair. This revealed that only two sets of sequences were 100% identical. Both were observed within the group of E type sequences, one representing two and the other representing four independent samples. Therefore, 35 unique concatenated sequencing types (2 A-type, 8 B-type, and 25 E-type; Additional file 2: Table S2) were available for further analysis.

**Figure 1:**
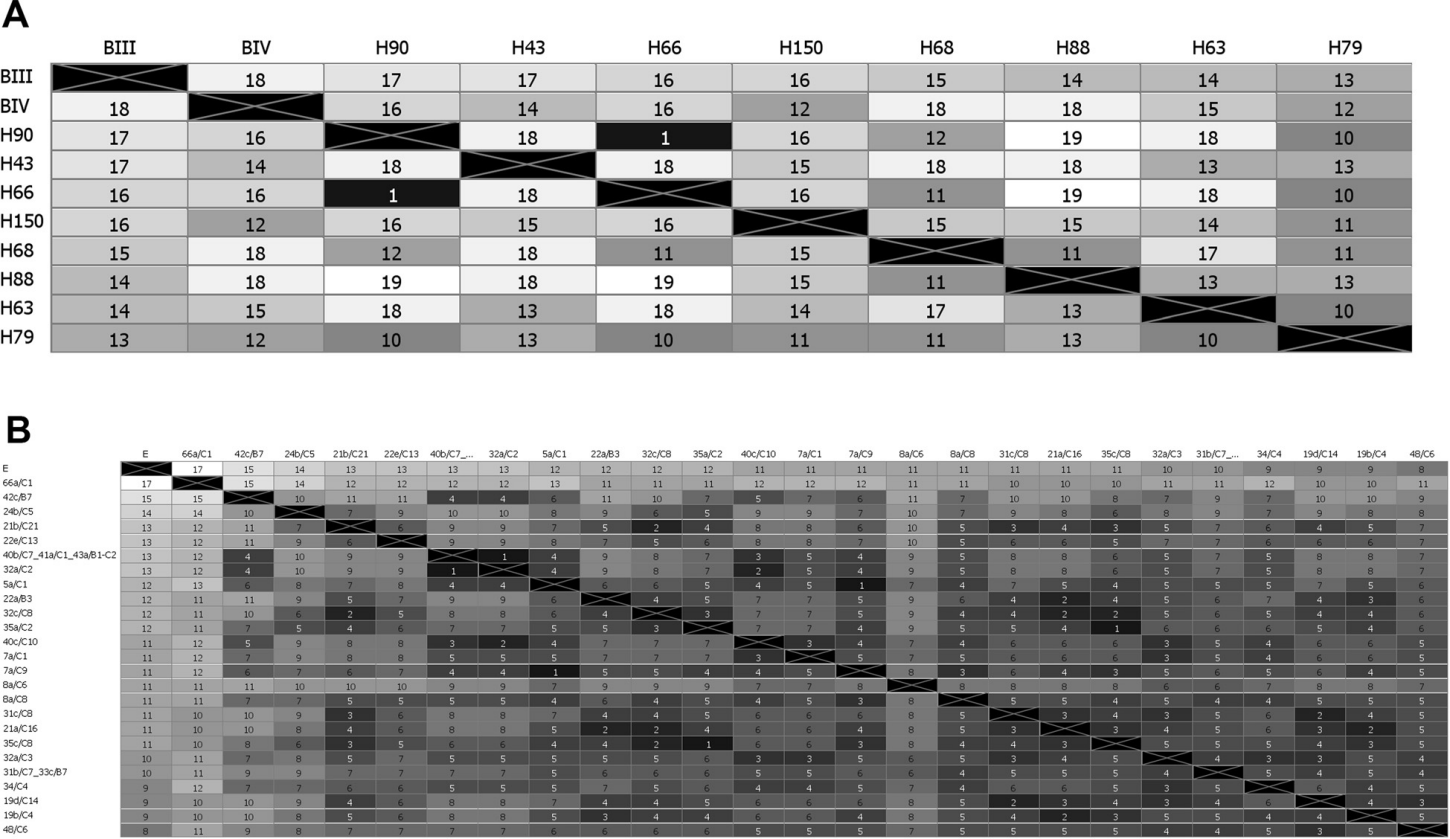
**Relatedness of assemblage B and E sequencing types using distance matrix analysis.** The sequencing fragments of samples with the complete information at all three gene loci were concatenated in the order of the *tpi-bg-gdh* sequences*.* The resulting sequencing fragments (1358 bp) were subsequently aligned using ClustalW. Shown are the deduced distance matrices for assemblage B **(A)** and assemblage E **(B)** sequencing types. Numbers and heatmap indicate nucleotide residues not identical between two sequences. We revealed only two complete type A sequences and these were excluded from the analysis. **(A)** Eight unique type B sequences could be analyzed. **(B)** 25 unique sequences (of a total of 29 type E sequences) were retrieved and analyzed. Samples 31b/C7 and 33c/B7 were identical as well as samples 40b/C7, 41a/C1, 43a/B1 and 43a/C2.

**Figure 2:**
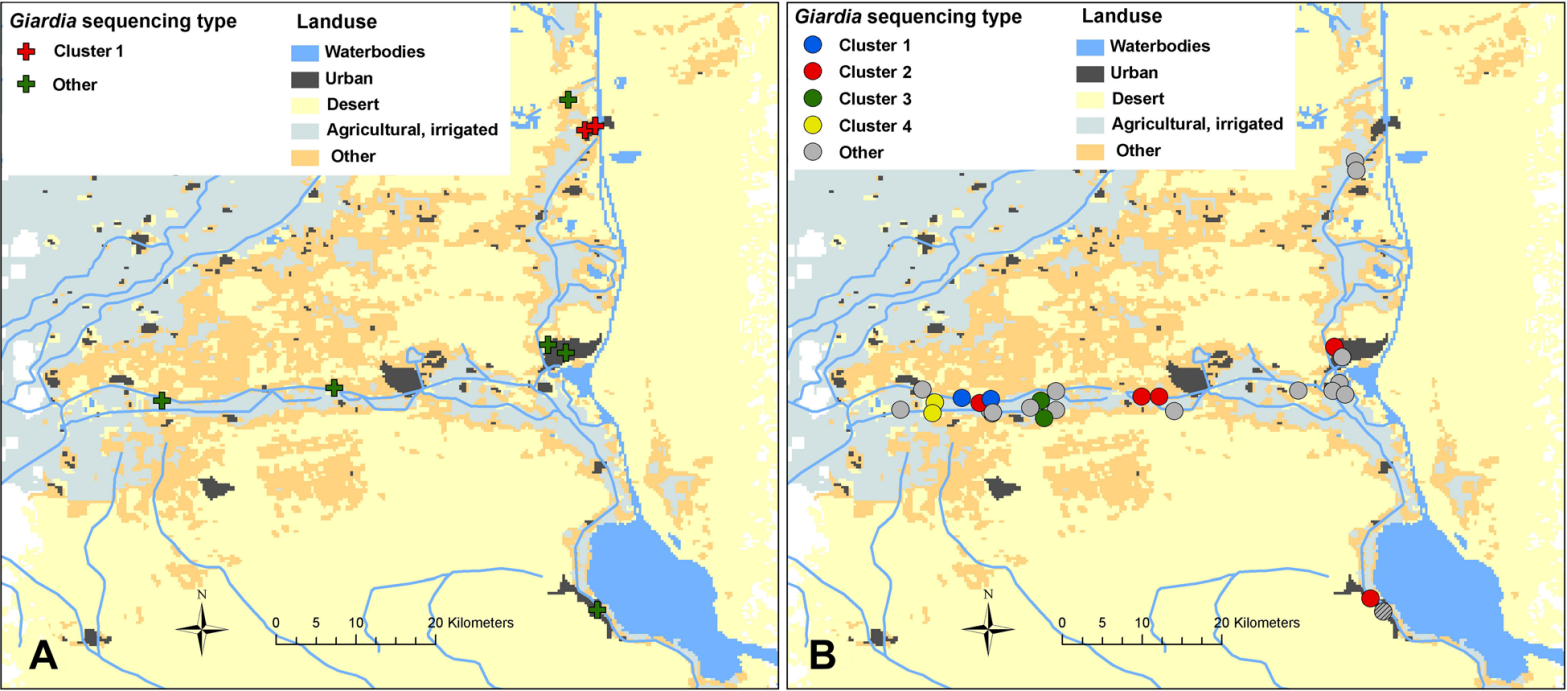
**Spatial distribution of samples containing assemblage B and E type parasites.** Clusters of concatenated B and E type sequences were defined based on sequence identity, i.e. sequences that were identical or that differed in maximally one base pair were combined in one cluster. The spatial distribution of sequence types was visualized on a map of the study region. **(A)** Analysis of the 8 B type sequences revealed one cluster with 2 sequences that differed in one base pair (samples H66 and H90). **(B)** Analysis of 29 E type sequences revealed 4 clusters. Cluster 1 comprising of the identical sequences of samples 31b/C7 and 33c/B7. Cluster 2 comprising of the 4 identical sequences of samples 40b/C7, 41a/C1, 43a/B1 and 43a/C25, and of sequence of sample 32a/C2 that differed in one base pair. Cluster 3 comprising of sequences of samples 5a/C1 and 7a/C9 that differ in one base pair. Cluster 4 comprising of sequences of samples 35a/C2 and 35c/C8 that differ in one base pair. Note that sample 40c/C10 (shaded grey area) differed only in three base pairs from sample 40b/C7 of cluster 2.

Analysis of the pairwise comparisons of unique sequences belonging to the two larger groups showed that B-type sequences differed in 12 to 18 residues over their length of 1358 bp from the chosen reference BIII and BIV fragments (Figure 1A). For E type sequences, this range was 8–17 residues different to the reference E fragments (deduced from respective genes of the sequenced isolate P15; Figure 1B). Within the groups of Ismailia samples, B sequencing types differed on average in 14 residues and E types in 6 residues. These values are consistent with the notion that intra-assemblage heterogeneity of B type isolates is higher than that of E or A type parasites [17].Data on isolate genetic relatedness was integrated next with information on the geographic origin of the samples and projected onto a map of Ismailia province (Figure 2). Samples containing parasite DNA that was characterized by either an identical or near identical (1 of 1358 bp different) concatenated sequencing type were spatially related in 4 of 5 cases. The two human samples with a B sequencing type differing in a single base pair (sample pair number H66 and H90) were collected from children living in the same locality (Figure 2A). Similarly, all but one group of animal samples that contained E sequencing types that were genetic pairs (i.e. showed complete sequence identity or differed by only one base pair) were also geographic neighbors (Figure 2B; Clusters 1, 3, and 4). The distance between sample pairs that belonged to either of these assemblage B or E clusters was significantly shorter from the average distance calculated for all possible sample pairs (1.6 km vs 27.5 km; p < 0.01; two sided Student’s T Test). Moreover, two of the samples belonging to cluster 2 (representing 5 samples, 4 with identical sequencing type and one with a single base pair difference; Figure 2B) originated from the same herd. Another one, 40b/C7, differed in only 3 base pairs from sample 40c/C10 - the geographic neighbor, but displayed on average 7 bp difference with the sequencing types of other samples. Therefore, sequence information, regarding even just these three typing loci, provided a high enough resolution to identify subpopulations of parasites that seemed to be linked epidemiologically.

## Discussion

Understanding the epidemiology of giardiasis and estimating the zoonotic potential of its etiologic agent is necessary for rational disease management, particularly in regions of high prevalence in animals and/or humans.

In this study the epidemiology of *G. duodenalis* infection in Ismailia province, Egypt was investigated. Previous studies on *Giardia* infections in Egypt and the Middle East have concentrated on detecting and analyzing human infections [31-39]. Depending on the study, a prevalence of 10-40% was reported. Here, this knowledge is complemented by providing prevalence estimates, genetic characterization and spatial distribution data of *G. duodenalis* assemblages detectable in samples from diarrheic children and animals from this geographic area. Overall prevalence in livestock was 53%, while *G. duodenalis* was found in one of five children with diarrheal symptoms. While our typing results confirmed previous reports [13,40] that zoonotic transmission from farm animals can occur, they also show that this is an infrequent event, even in an area of high *Giardia* prevalence in livestock and presumably low hygiene conditions. Consistent with this result, it was found that drinking tap water but not contact with animals was associated with increased risk for children to contract the infection. Thus, a dominant anthropogenic cycle is assumed that may be fuelled by low microbiological quality of water supply. Improving the latter will therefore be most likely efficient in reducing the burden of human infection in these sites.

The prevalence of 21% of *Giardia* infection in children with diarrheal symptoms sampled here was unexpectedly low. A report by Mahmoud *et al*. on *G. duodenalis* infection in infants from Bilbeis (a neighboring locality of our study area) showed that during the first year of life a child contracts on average 4.5 infections that last on average 7.2 weeks [41]. Based on this, a prevalence of 60-70% was expected in children. Mahmud *et al*. also noted that children with diarrhea are much less likely to be tested positive for *G. duodenalis*, however, they did not elaborate on this in their report*.* Using the figures from the latter report for the diarrheal subgroup, a prevalence of 25% can be calculated, i.e. a number close to the 21% observed in the symptomatic group reported here. Data from at least three other studies suggest an inverse correlation between *G. duodenalis* infection in children and diarrheal symptoms [5,42,43]. This may be explained by a protective effect of *Giardia* colonization against unrelated causes of diarrhea; alternatively it may reflect a lower rate of parasite detection in symptomatic cases if diarrhea is viewed as a response of the host organism to reduce parasite burden.

The Bilbeis study [41] as well as others [5,44,45], also suggested that breast feeding has a preventive or protective effect against *G. duodenalis* infection. Thus, one would expect to detect lower parasite burdens in breast-fed infants. This is corroborated by the trend that was observed in the pediatric population investigated here. Children below 1 year of age, which in this area were likely to be breast-fed, displayed a higher prevalence based on real-time PCR but lower abundance of *G. duodenalis* compared to older children. Future work will have to substantiate this observation.

Gastrointestinal infections may predispose for co-infections. A possible positive association of *Cryptosporidium* and *Giardia* infections has been reported in some earlier studies [46-48], but most reports investigating possible co-infections with these two parasites did not find such an association [49-51]. In the present study a positive association between *Giardia* and *Cryptosporidium* infections in ruminants was observed; this may be due to a high rate of concomitant contamination of the water resources with both parasites.

Molecular typing of pathogens has enabled highly informative insight into the epidemiology of many infections [52]. Implementation of nucleic acid-based detection and typing methods over the past decade has led researchers to better understand the complexity of the *Giardia* genus including the parasite population relevant to human and veterinary health [15,18]. Before discussing our findings in this regard, some general comments about interpreting typing data in this field are warranted as this remains difficult [18].

Firstly, the parasites have two equivalent nuclei with full diploid chromosomal content and a single parasite could theoretically present up to four alleles of a single copy gene. Since parasites of only a minority of assemblages can be cultured and cloned with reasonable success, typing for the majority of samples relies on DNA prepared from cysts and typing of single cysts is currently not a realistic option. As a consequence, “mixed” sequencing results cannot unambiguously be assigned to mixed infection (which seems more probable in case of inter-assemblage ambiguities; cf. [16,18]) rather than to infection with heterozygotic parasites (more likely in case of “double peaks” and intra-assemblage ambiguities). Secondly, current PCR-based locus-specific typing assays show significant differences in sensitivity and bias in efficiency of amplifying a given assemblage [18]. This was also observed in our study. For example, locus-directed, assemblage A and E specific PCR assays displayed a lower sensitivity than the non-assemblage-specific assays and the *tpi* locus directed general PCR assay likely introduced a typing bias such that assemblage A type sequences were more readily detected. Out of 11 animal samples, which were typed as assemblage A, only two could be typed at other loci and, when successful, these were identified as type E amplicons. Re-testing these two samples with assemblage A and E-specific *tpi*-directed assays showed the same result. In view of all these caveats, we interpret the obtained typing data as providing operational rather than categorical classification of the parasite DNA.

In agreement with others [16], all sequencing types with assignments to more than one assemblage were considered as the result of mixed infections. Typically these typings were less robust either because fragments of only one locus could be amplified or because repeated analyses using more assemblage-specific typing assays failed possibly due to the lower sensitivity. In contrast, typing results showing intra-assemblage ambiguities were much more reliable, i.e. they were reproducible within a sample and identical patterns were repeatedly observed in independent samples. Although sexual recombination in *Giardia* may occur [53-55], at the population level this is thought to be rare and mostly to involve selfing [16]. Hence, it is reasonable to assume that particular sequencing types with data available for all three loci assigned to only one assemblage reflect distinct, identifiable and traceable isolates. Consistent with this, a closely related sequencing type was a good predictor for a similar geographic origin. Therefore, it is proposed that at least for assemblages B or E, intra-assemblage sequence differences are large enough to allow detection of epidemiological links even with the currently most widely used typing scheme that is based on just three loci. Consequently, infections due to parasites with identical typing fragments but for which respective samples were collected from hosts living more distantly from each other *may* therefore be linked by factors that had not been recorded here, such as animal, food or manure trading.

## Conclusion

*G. duodenalis* in Ismailia province was commonly found in livestock and children. Infection and diarrheal symptoms were not positively correlated; rather this relationship was inversed in children. Findings from genetic characterization indicate that infection cycles in humans and ruminants are largely separated. Thus, the risk of zoonotic infection emanating from cattle and buffalo is negligible from an epidemiological point of view. Distinct circulating parasite subpopulations were traceable by sequence typing. This suggested that the methodology is suitable for epidemiological studies on occurrence and spreading of *G. duodenalis* in humans and animals. This may also prove useful to monitor the effects of strategies that aim at interfering with transmission.

## Competing interests

The authors declare that they have no competing interests.

## Authors’ contributions

YAH performed the experiments, analyzed the data, and wrote the manuscript; CK and TA designed and supervised the study, analyzed the data and wrote the manuscript; HW perfomed the spatial distribution analysis; JK, KN, and GvH contributed reagents and wrote the manuscript; KHZ performed the initial study desing for sample collection and wrote the manuscript. All authors read and approved the final version of the manuscript.

## Supplementary Material

Additional file 1 Analysis of heterozygous sequences and ambiguous single nucleotide polymorphisms. Click here for file

Additional file 2 **Summary of genetic characterization of *Giardia *** **samples by PCR.** Click here for file
