# Supplementary material for: Epidemiology of Giardia duodenalis infection in ruminant livestock and children in the Ismailia province of Egypt: insights by genetic characterization
Source: Parasit Vectors. 2014 Jul 11;7:321. doi: 10.1186/1756-3305-7-321 (PMC4230635; doi:10.1186/1756-3305-7-321)
Supplement: Additional file 1 — Analysis of heterozygous sequences and ambiguous single nucleotide polymorphisms. [file 1756-3305-7-321-S1.pdf]

**Samples ID / Genbank accession #**	**Ref. seq**	**Position**																
		**96**	**130**	**159**	**167**	**171**	**208**	**225**	**230**	**232**	**238**	**248**	**267**	**280**	**297**	**376**	**400**	**435**
**GIU57897**	**AII**	**A**	**C**	**C**	**A**	**C**	**A**	**G**	**G**	**C**	**A**	**A**	**T**	**A**	**G**	**C**	**T**	**T**
**FJ560569**	**AI**	.	**T**	.	.	.	.	.	.	.	.	.	.	.	.	.	**C**	.
18d/B13		.	.	.	T	.	.	.	.	M	.	R	Y	.	.	.	.	.
14b/C5		.	.	.	.	Y	R	.	.	.	R	.	.	R	.	.	.	Y
10/C11-H67-H99-H77-H141-H64		.	.	.	.	.	.	.	.	.	.	.	.	.	.	.	.	.
54b/C7		.	T	.	.	.	.	.	.	.	.	.	.	.	.	.	C	.
18a/C5		.	T	T	.	.	.	.	.	.	.	.	.	.	.	Y	C	.
42a/C3		.	T	.	.	.	.	.	.	.	.	.	.	.	A	.	C	.
18d/B14-4/C2		.	.	.	.	.	.	.	.	.	.	.	.	.	.	.	C	.
8b/C11		G	.	.	.	.	.	C	T	.	.	.	.	.	.	.	C	.

		**40**	**52**	**57**	**92**	**130**	**163**	**166**	**169**	**193**	**199**	**210**	**238**	**341**	**244**	**256**	**298**	**313**	**437**	**439**	**484**
**L02116 (GS/M)**	**BIV**	**A**	**G**	**A**	**C**	**C**	**G**	**T**	**T**	**A**	**C**	**A**	**A**	**C**	**C**	**G**	**A**	**C**	**G**	**T**	**A**
**AY228628**	**BIII**	**G**	.	.	**T**	.	.	**C**	**C**	.	.	**G**	.	.	.	.	.	.	.	.	.
H88		G	.	.	.	.	A	C	Y	.	.	G	.	.	T	R	.	.	.	.	.
H68		G	.	.	.	.	A	C	C	.	.	G	.	.	T	A	.	.	.	.	.
H33		G	.	.	.	T	.	C	C	G	.	G	.	.	.	.	.	.	A	.	.
H43		.	.	.	T	.	.	Y	C	.	Y	G	.	Y	.	.	.	.	.	Y	R
H63		.	.	.	.	Y	.	C	C	R	.	R	R	.	.	.	.	.	.	.	R
H150		.	A	.	T	.	A	C	C	.	.	G	.	.	.	.	G	.	.	.	.
H66-H90		G	.	.	Y	.	.	C	C	.	.	G	.	.	.	.	.	Y	.	.	.
H19		.	.	G	T	.	.	C	C	.	.	G	.	.	.	.	.	.	.	.	.
H79		.	.	.	.	.	.	C	C	.	.	G	.	.	.	.	.	.	.	.	.
H86		G	.	.	.	.	.	.	C	.	.	G	.	.	.	A	.	.	.	.	.

		**52**	**73**	**94**	**101**	**110**	**145**	**160**	**192**	**265**	**340**	**363**	**364**	**390**	**490**	**509**
**EU781026 (P15)**	**E**	**G**	**C**	**C**	**C**	**A**	**A**	**A**	**C**	**C**	**G**	**G**	**G**	**G**	**G**	**G**
42c/B9		.	T	T	.	G	R	.	.	.	.	A	T	.	A	.
7a/C9-32a/C2-40b/C7-40c/C10-41a/C1-43a/B1-43a/C2-44b/C5-8a/C8-5a/C1		.	T	T	.	G	.	.	.	.	.	A	T	.	A	.
42c/B7		.	T	T	.	G	.	.	.	.	.	A	T	.	A	R
7a/C1		.	T	Y	.	G	.	.	.	Y	.	A	T	.	A	.
24b/C5		R	T	.	.	G	.	.	.	.	.	A	T	.	A	R
8a/C6		A	T	.	.	G	.	R	.	.	.	R	T	.	A	.
66a/C1-63b/C4-C8b/C11		A	T	.	.	G	.	G	.	.	.	A	K	.	A	.
9a/C1		A	T	.	.	G	.	G	T	.	.	A	T	.	A	.
17/C2		A	T	.	.	G	.	G	.	.	.	A	T	A	A	.
22a/B3-35c/C8-48/C6-31b/C7-33c/B7		.	T	.	.	G	.	.	.	.	.	A	T	.	A	.
22e/C13		.	T	.	T	G	.	.	.	.	.	A	T	.	A	.
35a/C2		.	T	.	.	G	.	.	.	.	.	A	T	.	A	R
31a/C1		.	Y	.	.	G	.	.	.	.	R	A	T	.	A	.
19b/C4-34/C4-53b/C5		.	C	.	.	G	.	.	.	.	.	A	T	.	A	.
21a/C16-32c/C8-34/C18		.	T	.	.	G	.	.	.	.	R	A	T	.	A	.
31c/C8-32a/C3-21b/C21-19d/C14		.	T	.	.	G	.	.	.	.	A	A	T	.	A	.

Note: Incomplete sequences or sequences with evidence for mixed infections were excluded

Samples ID / Genbank accession #	Ref. seq	Position						
		**100**	**123**	**362**	**370**	**383**	**390**	**508**
**HM165227**	**AII**	**C**	**T**	**C**	**T**	**A**	**G**	**T**
**HQ179590**	**AI**	.	.	.	.	.	.	**C**
H67-H99-H64		.	.	.	.	.	.	.
H77		T	.	.	.	.	.	.
19a/C3		.	C	.	.	G	A	C

Samples ID / Genbank accession #	Ref. seq	112	127	136	175	187	256	259	268	271	325	460	487	523
**EU014391 (GS/M)**	**BIV**	**T**	**G**	**A**	**G**	**T**	**T**	**T**	**C**	**T**	**C**	**C**	**C**	**G**
**AY072725**	**BIII**	**C**	.	**G**	**A**	.	**C**	**C**	.	**C**	.	.	.	.
H66-H90		Y	.	G	A	.	Y	C	Y	C	Y	.	.	.
H43		.	.	G	A	C	C	C	.	C	.	.	.	.
H88		C	.	G	A	.	C	C	.	C	.	.	Y	W
H68		C	.	G	A	.	C	C	.	C	.	.	T	A
H63-H69-H86		C	.	G	.	.	C	C	.	C	.	.	.	.
H79		C	.	G	A	.	Y	C	.	C	.	.	.	.
H74		C	.	G	A	.	.	C	.	C	.	T	.	.
H150		C	A	G	A	.	.	C	.	C	.	.	.	.

Samples ID / Genbank accession #	Ref. seq	94	103	203	210	310	443	450	523	526
**AY072729 (P15)**	**E**	**G**	**C**	**C**	**C**	**C**	**G**	**T**	**G**	**C**
24b/C5		.	T	.	.	.	.	C	.	.
4/C5		A	T	.	.	.	.	C	.	T
6/C3		R	T	.	.	.	.	C	.	Y
19d/C11-21b/C19-21b/C21		.	.	.	.	.	.	C	A	.
35a/C2-35c/C8-32c/C8		.	.	.	.	.	.	C	.	.
19b/C4-21a/C16-22a/B3		.	.	.	.	.	.	Y	.	.
31c/C8		.	.	.	.	.	.	.	R	.
31a/C1-34/C4-32c/C9-42a/C3-10/C6-8b/C11-53b/C5-19d/C14		.	.	.	.	.	.	.	.	.
22e/C13		.	.	.	.	.	C	.	.	.
45/C5		.	.	Y	.	.	.	.	.	.
C8/4		.	.	.	T	.	.	.	.	.
66a/C1		.	.	.	Y	.	.	.	.	.
8a/C8		.	Y	.	.	.	.	.	.	.
31b/C7-33c/B7		.	Y	.	.	Y	.	.	.	.
7a/C9		.	T	.	.	.	.	Y	.	.
45/C5		.	T	.	.	.	.	.	.	.
5a/C1		.	T	.	.	Y	.	Y	.	.
8a/C6		.	T	.	.	Y	.	.	.	.
30c/B11-31a/C4-32a/C3-42c/B9-32a/C2-40b/C7-40c/C10-41a/C1-42c/B7-43a/B1-43a/C2-44b/C5-7a/C1		.	T	.	.	T	.	.	.	.

Note: Incomplete sequences or sequences with evidence for mixed infections were excluded

Samples ID / Genbank accession #	Ref. seq	Position														
		63	78	110	126	174	200	213	246	273	276	282	327	380	408	426
**JQ700433**	**AII**	**C**	**C**	**T**	**T**	**C**	**A**	**C**	**C**	**C**	**C**	**G**	**G**	**A**	**C**	**C**
**AB692779**	**AI**	.	.	.	.	.	.	.	.	.	.	**T**	.	.	**T**	**T**
H99-H77		.	.	.	.	.	.	.	.	.	.	.	.	.	.	.

Samples ID / Genbank accession #	Ref. seq	63	72	78	79	84	101	114	162	201	206	234	252	345	351	366	375	402	411	417
**GL50581_4496 (GS/M, in Giardiadb.org)**	**BIV**	**G**	**G**	**T**	**C**	**C**	**C**	**T**	**C**	**T**	**G**	**C**	**C**	**T**	**C**	**T**	**C**	**C**	**C**	**A**
**AF069059**	**BIII**	.	.	.	.	.	.	**C**	**T**	.	.	**T**	**T**	**C**	.	**C**	.	.	.	**G**
H69		.	.	.	.	.	.	C	T	.	.	T	T	C	T	C	.	.	.	S
H86		.	.	.	.	.	.	Y	Y	.	.	Y	Y	Y	Y	C	Y	.	.	.
H87		C	A	C	.	.	T	.	T	.	A	.	.	.	.	.	.	.	.	.
H74		.	.	.	T	.	T	.	T	.	.	T	.	.	.	.	.	.	.	.
H66		.	.	.	.	T	.	C	.	.	.	.	.	.	.	Y	.	Y	.	.
H90		.	.	.	.	T	.	Y	.	.	.	.	.	.	.	Y	.	Y	.	.
H79		.	.	.	.	.	.	.	Y	Y	.	.	.	.	.	Y	.	Y	.	.
H63		.	.	.	.	.	.	.	Y	.	.	.	.	.	.	C	.	.	.	R
H88		.	.	.	.	.	.	.	Y	.	.	Y	.	.	.	C	.	.	.	R
H68		.	.	.	.	.	.	.	T	.	.	.	.	.	.	C	.	.	.	.
H150		.	.	.	T	.	.	.	.	.	.	.	.	.	.	C	.	.	.	.
H43		.	.	.	.	.	.	.	.	.	.	.	.	.	.	C	.	.	T	.

Samples ID / Genbank accession #	Ref. seq	63	66	105	135	189	222	297	318	324	350	370	387	429	432	438	444
**AY178741 (P15)**	**E**	**A**	**C**	**T**	**G**	**C**	**G**	**C**	**C**	**C**	**C**	**G**	**G**	**A**	**C**	**C**	**C**
20d/C16		**G**	.	.	.	.	.	.	.	.	.	.	.	.	.	.	.
36c/C10-19d/C14		.	.	.	.	.	.	.	.	.	.	.	.	.	.	.	.
48/C6		.	.	.	.	.	.	.	.	.	.	.	.	.	.	.	T
34/C18		.	.	.	A	.	A	.	.	.	Y	.	R	.	.	.	T
36a/C1		.	.	.	A	.	A	.	.	.	.	.	.	.	.	.	T
31a/C4-31b/C7-33c/B7		.	.	.	.	.	.	.	.	.	.	.	R	.	.	.	.
19b/C4-21a/C16-30c/B11-31c/C8-32a/C3-32c/C9-8a/C6-10/C6-45/C5-51a/B2-7a/C1-55c/B10		.	.	.	.	.	.	.	.	.	.	.	A	.	.	.	.
42a/C3		.	.	.	.	.	.	.	Y	.	.	.	A	.	.	.	.
66a/C1		.	.	.	.	.	R	Y	.	Y	.	.	A	.	Y	Y	.
4/C5		.	Y	.	.	.	R	.	.	.	.	.	A	.	.	.	.
6/C3		.	.	Y	.	.	A	.	.	.	.	.	A	.	.	.	.
41a/C1		R	.	.	.	.	R	.	.	.	Y	.	A	.	.	.	.
7a/C9-22a/B3-35a/C2-35c/C8-5a/C1		.	.	.	.	.	.	.	.	.	Y	.	A	.	.	.	.
22e/C13		.	.	.	.	.	.	.	.	.	T	.	A	G	.	.	.
34/C4-34/C9-8a/C8-21b/C21-32c/C8-H31		.	.	.	.	.	.	.	.	.	T	.	A	.	.	.	.
2a/C2		.	.	.	.	.	.	.	.	.	T	A	A	.	.	.	.
24b/C5		.	.	.	.	T	.	.	.	.	T	.	A	.	.	.	.
40b/C7		R	.	.	.	.	R	.	.	.	Y	.	A	.	.	.	.
43a/B1-43a/C2		R	.	.	.	.	R	.	.	.	Y	.	A	.	.	.	.
32a/C2		G	.	.	.	.	R	.	.	.	Y	.	A	.	.	.	.
40c/C10		G	.	.	.	.	.	.	.	.	.	.	A	.	.	.	.
17/C2		C	.	.	.	.	.	.	.	.	.	.	A	.	.	.	.
27a/C1		R	.	.	.	.	R	.	.	.	.	.	A	.	.	.	.
42c/B7		C	.	.	.	.	A	.	.	.	Y	.	A	.	.	.	.

Note: Incomplete sequences or sequences with evidence for mixed infections were excluded
